# Antimicrobials in dog‐to‐dog bite wounds: A retrospective study of 1526 dog bite events (1999‐2019)

**DOI:** 10.1111/jvim.16574

**Published:** 2022-11-18

**Authors:** Nicole J. Kalnins, Justine S. Gibson, Allison J. Stewart, Catriona Croton, Sarah L. Purcell, Bandula Rajapaksha, Mark Haworth

**Affiliations:** ^1^ School of Veterinary Science University of Queensland Gatton Queensland Australia; ^2^ School of Sciences, Faculty of Health, Engineering and Sciences University of Southern Queensland Darling Heights Queensland Australia

**Keywords:** antimicrobial stewardship, antimicrobial susceptibility, bacteriology, canine, trauma

## Abstract

**Background:**

Although dog‐to‐dog bite wounds (DBW) are common, few studies worldwide have evaluated antimicrobial usage patterns or appropriateness of use.

**Objectives:**

Report frequency and results of DBW cultures, including antimicrobial susceptibility patterns. Determine the most commonly prescribed antimicrobials and their appropriateness for the treatment of DBW, and if antimicrobial importance is associated with wound severity, clinic type or year.

**Animals:**

One thousand five hundred twenty‐six dog bite events involving 1436 dogs presenting with DBW from 3 Australian university clinics from 1999 to 2019.

**Methods:**

Retrospective study. Medical records were reviewed for presenting signs, culture and susceptibility testing, antimicrobial treatment, and outcome. A partial proportional odds model was used to determine if use of higher importance antimicrobials was associated with wound severity, clinic, or year.

**Results:**

Antimicrobials were prescribed in 88.1% (1344/1526) of DBW. Amoxicillin‐clavulanic acid was prescribed in 73.4% (1121/1526) of dogs, followed by first‐generation cephalosporins, 18.1% (277/1526). Of a total of 1647 antimicrobial prescriptions, underdosing occurred in 13.4% for AMC (220/1647) and 26.1% (81/310) of dogs prescribed first generation cephalosporins. There was an association between the increased use of high‐importance antimicrobials and wound severity (*P* < .001), antimicrobial polytherapy (*P* < .001) and year (*P* < .001). The odds of the clinic with specialists prescribing high‐importance antimicrobials compared to those of medium importance for DBW was 82% less than that of a semi‐rural, mixed and general practice. Culture and susceptibility (C&S) testing was performed in 1.8% of dogs.

**Conclusion and Clinical Importance:**

Empirical use of amoxicillin‐clavulanic acid was common for DBW. Increasing wound severity was associated with greater use of high‐importance antimicrobials. While C&S testing was rarely performed, routine susceptibility profiles are recommended to optimize antimicrobial stewardship.

**Abbreviations**
C&S: culture and susceptibility
DBW: dog‐to‐dog bite wound
DFW: dog fight wound
ER: electronic record
IQR: interquartile range
OR: odds ratio
SEQ: South East Queensland
SQL: structured query language
UQ: University of Queensland, Australia

## 1 INTRODUCTION

Dog‐to‐dog bite wounds (DBW) commonly present to veterinary clinics and emergency centers, accounting for 10% to 15% of trauma cases globally.^1^, ^2^, ^3^, ^4^ Despite this high incidence, there is limited experimental and clinical evidence to support scientific recommendations on antimicrobial treatment of these wounds.^3^, ^5^ DBW are contaminated from bacteria found in the attackers' mouth, commensals from the victims' skin and from the environment.^5^, ^6^, ^7^, ^8^ Few studies have reported the bacteria which contaminate and infect DBW. The most common isolates previously reported were *Staphylococcus pseudintermedius*, *Enterococcus* spp., *Escherichia coli* and *Pasteurella multocida*.^4^, ^5^, ^7^, ^9^, ^10^, ^11^, ^12^, ^13^, ^14^

Human medical studies have found that antimicrobials are not required prophylactically in dog bite wounds, except in high‐risk cases.^15^, ^16^ In veterinary medicine, prophylactic antimicrobial therapy is widely used in DBW and is considered 1 of the mainstays of treatment.^5^ In addition to increased costs and the risk of adverse effects, inappropriate antimicrobial use could contribute to antimicrobial resistance.^5^ Limited experimental and clinical evidence currently exists to permit recommendation of appropriate empirical antimicrobial therapy of DBW.^5^, ^7^ The Australian Infectious Disease Advisory Panel (AIDAP), British Small Animal Veterinary Association (BSAVA) and the University of Melbourne's Asia Pacific Centre for Animal Health and the National Centre for Antimicrobial Stewardship (APCAH) have produced guidelines for the treatment of DBW which recommend antimicrobials for animals which are systemically unwell, have diffuse tissue involvement, potential joint involvement or are immunocompromised.^17^, ^18^, ^19^ Empirically, amoxicillin or amoxicillin‐clavulanic acid (AMC) is recommended, with additional antimicrobials prescribed based on culture and susceptibility (C&S) results.^2^, ^5^, ^12^, ^20^ However, a recent study demonstrated that C&S of DBW by veterinarians is rarely performed.^21^

Currently there is limited published data on the most cultured organisms from DBW in South East Queensland (SEQ). Data reporting antimicrobial susceptibility patterns for bacteria isolated from SEQ DBW will improve first choice antimicrobial selection, thereby reducing the rate of development of antimicrobial resistance, improve treatment success and outcomes. The aims of this study were to: 1. determine the most common antimicrobials prescribed to treat DBW and if the importance of the antimicrobial prescribed, as defined by the Australian Strategic and Technical Advisory Group of Antimicrobial Resistance (ASTAG),^22^ was associated with wound severity, clinic, or year and 2. determine the frequency of antimicrobial underdosing when used to treat DBW. The frequency and results of culture of DBW in SEQ, including antimicrobial susceptibility patterns was also investigated.

## 2 METHODS

A search of electronic records (ERs) of dogs presenting to 3 teaching hospitals from the same university from 1999 to 2019 was performed using the terms: “DBW,” “dog fight,” dog‐fight‐wound, “DFW,” “dog attack,” and “dog bite.” Clinic A was an urban, specialist referral/general practice hospital with a database extending from December 2002 until December 2013. Clinic B was a semirural, specialist referral/general practice hospital with a database extending from October 2011 to present day and Clinic C was a semirural, mixed and general practice clinic with a database extending from December 1999 to the present day. Consultations within 1 month of an identified DBW consultation were also extracted to ensure inclusion of reexamination.

**FIGURE 1:**
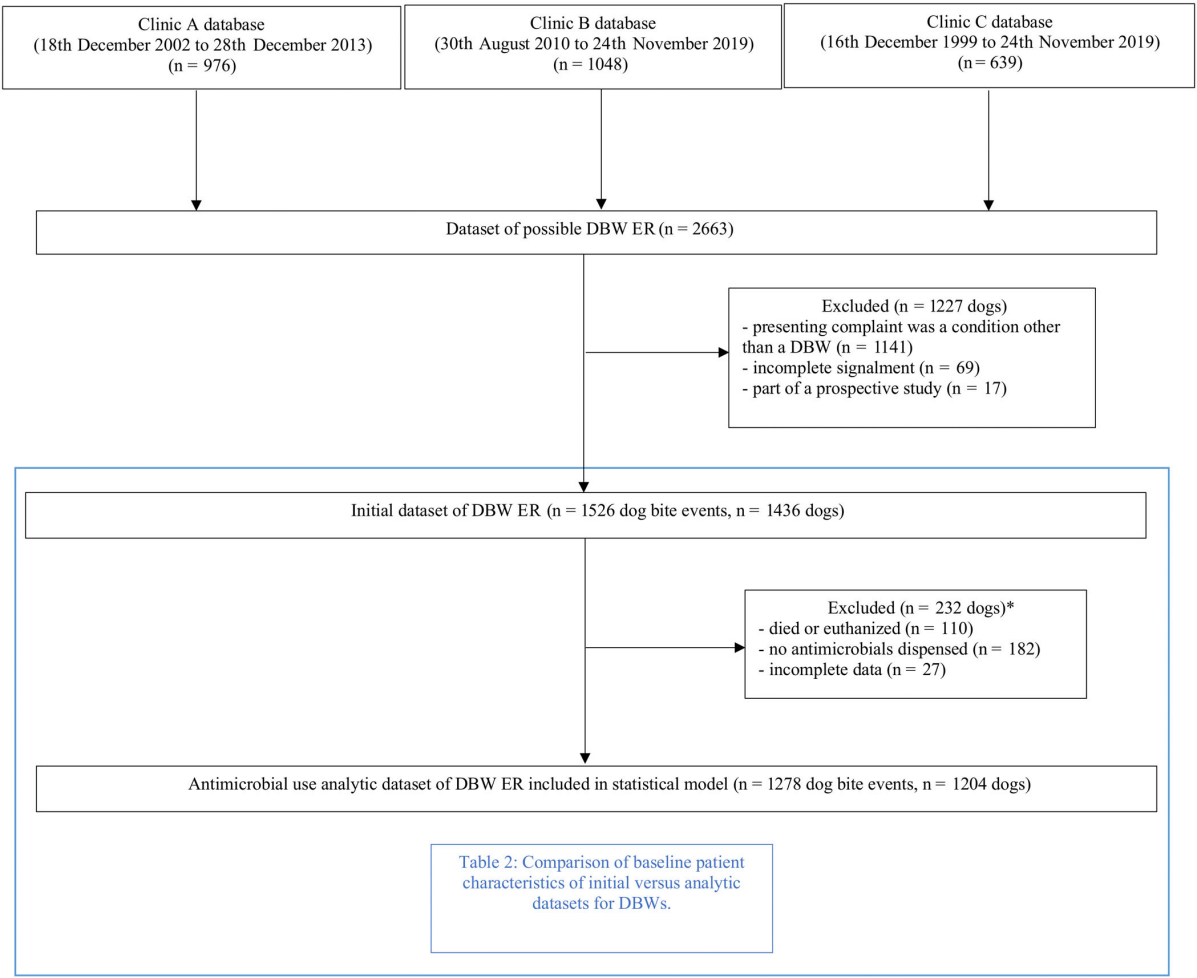
Flow chart showing creation of initial and analytic datasets and reasons for exclusion of possible DBWs presenting to Clinics A, B and C from 1999 to 2019. (DBW, dog‐to‐dog bite wound; ER, electronic record). *Eighty‐seven dogs had more than 1 factor for exclusion

The ERs were manually reviewed for signalment; time from injury to presentation; wound severity; type of antimicrobial prescribed; if a culture sample was taken; organism(s) cultured and their antimicrobial susceptibilities; hospitalization duration (days), complications and death. Electronic records were included in the study if the cause of injury was a known DBW as determined from the examination text field. The ERs were excluded if the injuries were not definitive for a DBW, incomplete signalment recorded or were part of a prospective DBW study; this resulted in the first dataset, “initial dataset” which was used for descriptive statistics. Subsequently, ERs for dogs which had no antimicrobials dispensed (including those who died or were euthanized at the initial consultation), or had incomplete treatment data were excluded, resulting in the second dataset, “analytic dataset” used for inferential statistical analysis (Figure 1). Animal ethics was approved by the University of Queensland Animal Ethics Committee.

A previously established grading system was used to categorize wound severity using the examination text.^5^, ^7^, ^12^ Grade 1 and 2 wounds were categorized as superficial wounds with partial thickness and full thickness laceration of the dermis, respectively. Grade 3 wounds were full thickness puncture wounds with penetration of the dermis without systemic illness. Grade 4 wounds were full thickness punctures or lacerations with avulsion of underlying tissues and dead space, underlying muscle trauma, possible penetration of a joint, abscess, or systemic illness. Grade 5 wounds were severe and included penetration into body cavities (abdomen, thorax) and open fractures. A laceration was defined as a wound >10 mm in length and a puncture as a wound <10 mm in length.

Antimicrobials prescribed were classified based on importance level as determined by ASTAG^22^ as low, medium and high‐importance (Table 1). In this study a prescription was considered a discrete course of antimicrobials dispensed for the duration of the DBW event in question. Injectable and oral antimicrobials are considered as a single prescription.

**TABLE 1:** Antimicrobial classification as defined by the Australian Strategic and Technical Advisory Group of Antimicrobial Resistance compared to the World Health Organization, relevant to the treatment of dog‐to‐dog bite wounds^22^, ^23^

Antimicrobial	ASTAG importance rating	WHO importance rating
Ampicillin/amoxicillin	Low	High priority, critically important
Amoxicillin‐clavulanic acid	Medium	High priority, critically important
Cephalosporin (1st and 2nd generation)	Medium	Highly
Cephalosporin (3rd generation)	High	Highest priority, critically important
Lincosamide	Medium	Highly
Metronidazole	Medium	Important
Penicillin	Low	Highly
Piperacillin/tazobactam	High	High priority, critically important
Quinolones/fluoroquinolones	High	Highest priority, critically important
Sulfonamides	Low	Highly
Tetracycline	Low	Highly

Samples for C&S testing were submitted to the same onsite university veterinary laboratory service in Clinics A and B. Clinic C submitted samples to a private external veterinary laboratory, however the culture methods were similar. All susceptibility testing was completed following the Clinical and Laboratory Standards Institute (CLSI) guidelines for disc diffusion testing.

Swabs submitted to IDEXX laboratories utilized matrix assisted laser desorption ionization‐time of flight mass spectrometry^i^ (MALDI‐TOF) for species identification. Susceptibility testing was done using Vitek‐2^j^ and results were interpreted by the Calibrated Dichotomous Sensitivity (CDS) method and the CLSI guidelines (CLSI, 2018).

### 2.1 Statistical analysis

For descriptive statistics, variables were summarized in accordance with their distribution and type, with normal variables as mean (SD), nonnormal variables as median (interquartile range) and categorical/binary data as proportion (%). To evaluate representativeness of the model sample, the initial and analytic datasets were assessed for statistically significant differences using *t*‐tests, Wilcoxon rank‐sum (Mann‐Whitney) test and chi‐square test for normal continuous, skewed continuous and categorical variables, respectively, with Bonferroni's correction for multiple comparisons.

A partial proportional odds model was fitted to the analytic dataset given the ordinal nature of the outcome variable antimicrobial importance, and the odds ratios (OR) represented study sample averaged effects with adjustment for clustering at the dog level. For the variables constrained to the proportional odds assumption, the OR were for being above a specified antimicrobial importance level compared with being at or below that importance level, with the assumption that the OR did not depend upon the importance level. The OR reported was the estimated effect of the given variable on the odds of being in the antimicrobial medium‐importance or high‐importance vs low importance; or of being in high importance vs low or medium‐importance levels. Two OR were reported for the unconstrained variables; the OR for being an antimicrobial of low‐importance vs high‐importance, and medium vs high‐importance. Likelihood ratio tests were used for assessing violation of the proportional odds assumption at a 0.1 level of significance.

Explanatory variables constrained to the proportional odds assumption included wound severity (grade 1‐5), type of therapy (monotherapy vs polytherapy), year of consultation category (1999‐2004, 2005‐2009, 2010‐2014, and 2015‐2019), clinic (A, B, C), season of consultation (Spring, Summer, Autumn, Winter), if C&S was performed, time from attack (<8 hours, ≥8 hours or unknown), duration of hospitalization (days), and the potential confounders of age (years), sex, neuter status, and weight (<10 kg, 10‐25 kg, >25 kg). Clinics A and C were constrained to the proportional odds assumption; this constraint was removed for Clinic B as it showed strong evidence of violating this assumption (*P* = .0008). This constraint was also removed for the year category of 2005‐2009 as it showed weak evidence of violation (*P* = .09). The baseline categories for the model are male, entire, Clinic C, weight group <10 kg, year category 1999‐2004, a single antimicrobial prescribed and time of attack being <8 hours.

The global proportional odds assumption was evaluated using a Wald test of the partial proportional odds model vs the multinomial logit model, with no evidence the assumption did not hold $\left(\chi^{2}\left(18\right)=24.7,P=.13\right)$. The ordinal outcome variable was dichotomized, and logistic regression model fitted to confirm similarity of regression coefficients to the proportional odds model. Cumulative sample logits were approximately linear.

Analyses were conducted in Stata version 16.1 (StataCorp. 2019. Stata Statistical Software: Release 16. College Station, Texas: StataCorp LLC). The significance level was set at .05, except for .1 for proportional odds assumption violation. The Reporting of studies Conducted using Observational Routinely‐collected health Data (RECORD) guidelines were used in the reporting of this study.^24^

## 3 RESULTS

A total of 2663 unique dogs were identified from the search of the 3 databases, with 1227 dogs subsequently excluded due to: presenting for reasons other than a DBW, incomplete ERs or enrolment in a simultaneous prospective study.^24^ The initial dataset consisted of 1526 dog bite events for 1436 individual dogs. Ninety dogs presented for multiple dog fights resulting in DBW. Multiple dog fights were considered as individual events when there was complete resolution of wounds from the first event and subsequent events were due to different fights and not reexaminations. For inferential analysis of antimicrobial use, a further 232 dogs for 248 unique DBW events were excluded due to death or euthanasia at initial consultation (n = 104), no antimicrobials dispensed (n = 101), and incomplete data (n = 27; Figure 1).

### 3.1 Signalment

The study cohort of 1436 dogs consisted of 762 males (53.1%), of which 469 (61.5%) were desexed, and 674 (46.9%) females with 476 (70.6%) desexed. The median age was 5 years (interquartile range [IQR] 3.0‐9.0) with a median weight of 18.7 kg (IQR 7.8‐27 kg; Table 2). There were 97 breeds represented: 1023 purebreds (71.2%) and 413 crossbreds (28.8%). The most common pure breeds were Staffordshire Bull Terriers (7.8%), Australian Cattle dogs (6.4%), Border Collies (4.2%), Fox Terriers (4.2%), Greyhounds (3.3%), and Jack Russell Terriers (3.2%). Other breeds constituted less than 3% each.

**TABLE 2:** Comparison of baseline dog characteristics of initial vs antimicrobial use analytic datasets for dogs presenting with dog‐to‐dog bite wounds from 1999 to 2019

	Initial dataset (n = 1, 436 dogs, 1526 DBW events)	Antimicrobial use analytic dataset^a^ (n = 1, 204 dogs, 1278 DBW events)	*P* value^b^	*P* value^c^
Median age, y (IQR)	5.0 (3.0‐9.0)	5.0 (3.0‐8.0)	.62	1.0
Sex			.83	1.0
Male	762 (53.1%)	637 (52.9%)		
Female	674 (46.9%)	567 (47.1%)		
Neuter			**<.001**	**<.001** *
Desexed	944 (65.7%)	825 (68.5%)		
Entire	492 (34.3%)	379 (31.5%)		
Median weight, kg (IQR)	18.7 (7.8‐27.0)	19.0 (8.1‐27.0)	.43	1.0
Wound severity^d^			**<.001**	**<.001** *
Grade 1	85 (5.6%)	22 (1.7%)		
Grade 2	164 (10.7%)	135 (10.6%)		
Grade 3	582 (38.1%)	552 (43.2%)		
Grade 4	621 (40.7%)	533 (41.7%)		
Grade 5	74 (4.9%)	36 (2.8%)		
Clinic			**.03**	.37
Clinic A	540 (37.6%)	462 (38.4%)		
Clinic B	521 (36.3%)	419 (34.8%)		
Clinic C	375 (26.1%)	323 (26.8%)		
Year^d^			**.002**	**.02** *
1999‐2004	136 (8.9%)	121 (9.5%)		
2005–2009	370 (24.2%)	310 (24.3%)		
2010–2014	506 (33.2%)	441 (34.5%)		
2015–2019	514 (33.7%)	406 (31.8%)		
Type of antimicrobial therapy^d^			**<.001**	**<.001** *
No antimicrobials	182 (11.9%)	0 (0%)		
Monotherapy	1048 (68.7%)	1001 (78.3%)		
Polytherapy	296 (19.4%)	277 (21.7%)		
Antimicrobial importance			**<.001**	**<.001** *
No antimicrobials	182 (11.9%)	0 (0%)		
Low	37 (2.4%)	34 (2.7%)		
Medium	1207 (79.1%)	1154 (90.3%)		
High	100 (6.6%)	90 (7.0%)		
C&S performed^d^	27 (1.8%)	26 (2.2%)	.61	1.0
Time of attack^d^			.13	1.0
<8 h	616 (40.4%)	528 (41.3%)		
>8 h	281 (18.4%)	226 (17.7%)		
Unknown	629 (41.2%)	524 (41.0%)		
Season of attack^d^			**.002**	**.03** *
Spring	348 (22.8%)	291 (22.8%)		
Summer	368 (24.1%)	289 (22.6%)		
Autumn	390 (25.6%)	326 (25.5%)		
Winter	420 (27.5%)	372 (29.1%)		
Median duration of hospitalization (days) (IQR)	0 (0‐1)	0 (0‐1)	.6	1.0

*Note*: Bolded values have a *p* value of 0.05 or less for ease or reading.

Abbreviation: IQR, interquartile range.

^a^ Antimicrobial use analytic dataset removed dogs which did not receive antimicrobials and had incomplete ERs.

^b^ Uncorrected *P* value.

^c^ *P* value corrected for multiple comparisons using Bonferroni method.

^d^ By unique consultation.

* Significant at the .05 level.

### 3.2 Wound severity

Of the 1526 DBW events, 85 dogs (5.6%) sustained grade 1 wounds, 164 (10.8%) grade 2, 582 (38.1%) grade 3, 621 (40.7%) grade 4, and 74 (4.8%) grade 5 wounds (Table 2).

### 3.3 Antimicrobials

**FIGURE 2:**
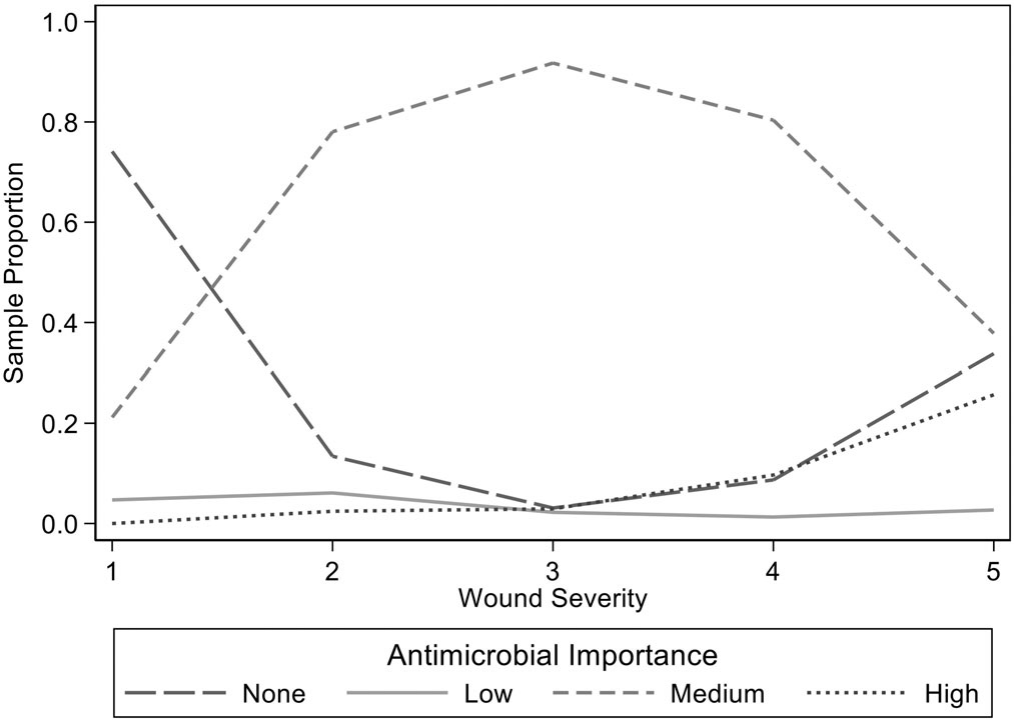
Antimicrobial importance class vs wound severity in 1436 dogs which presented for treatment for dog‐to‐dog bite wounds from 1999 to 2019

**FIGURE 3:**
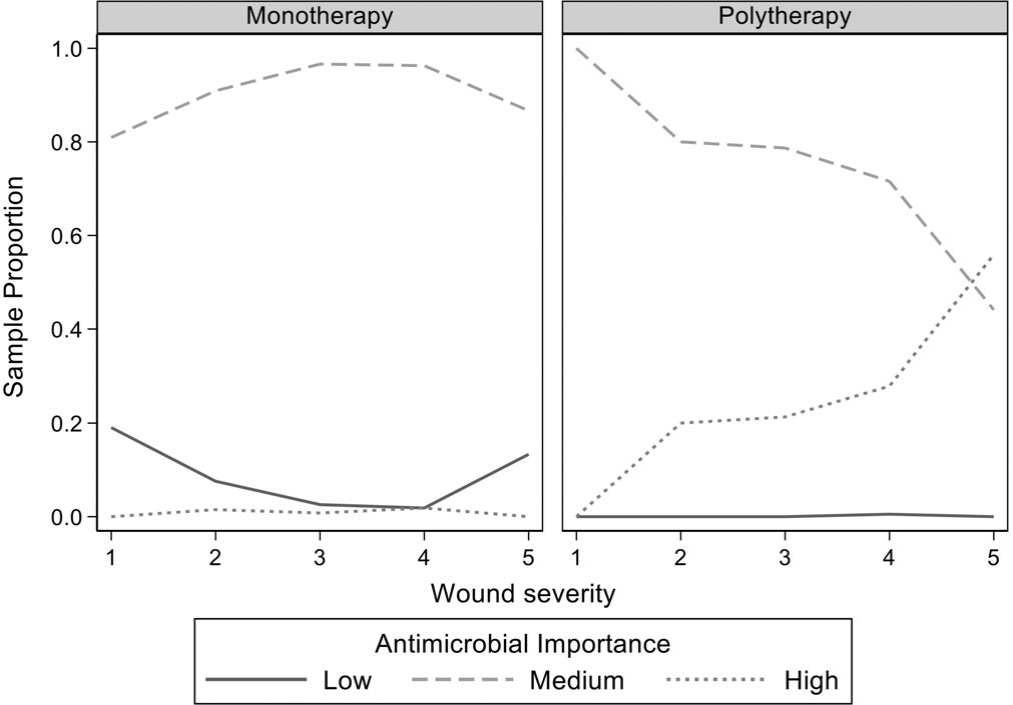
Sample proportions of antimicrobial importance class vs wound severity for monotherapy and polytherapy in 1204 dogs which presented for treatment of dog‐to‐dog bite wounds from 1999 to 2019. Dogs which received no antimicrobials were excluded

A total of 88.1% (1344/1526) dog bite events received at least 1 antimicrobial at presentation. No antimicrobials were prescribed to 182 dog bite events, due to not being deemed necessary (60.9%, 111/182) or the dogs were euthanized or died before antimicrobial administration (39.1%, 71/182). Seventy‐four percent (63/85) of dogs with grade 1 wounds and 13.4% (22/164) of grade 2 wounds did not receive antimicrobials (Figure 2). Thirty‐three percent (25/74) of dogs with grade 5 wounds did not receive antimicrobials, 21 of these dogs either died or were euthanized shortly after presentation. Of the dogs which received antimicrobial treatment, 78.0% (1048/1344) received monotherapy (dispensed antimicrobials from 1 class) and 22.0% (296/1344) received polytherapy (dispensed antimicrobials from more than 1 class; Table 2 and Figure 3).

AMC was the most prescribed antimicrobial with 73.4% (1121/1526) of dog bite events receiving parenteral, oral or both formulations, with 1647 prescriptions. First‐generation cephalosporins, cefazolin and cephalexin, were the second most frequently prescribed antimicrobial with 18.1% (277/1526) of dog bite events receiving parenteral, oral or both formulations with 310 prescriptions. The third‐generation cephalosporin, cefovecin, was prescribed to 0.8% (13/1526) of dogs and 7.6% (117/1526) of dogs received a fluoroquinolone (enrofloxacin) either PO, parenterally or both, 97% of which were prescribed as a component of polytherapy (Table 3).

**TABLE 3:** Antimicrobials and route of administration prescribed for 1526 dog bite events presenting for treatment between 1999 and 2019

Antimicrobial	Number of dogs	%
Amoxicillin‐clavulanic acid parenteral (SC)	621	40.7
Amoxicillin‐clavulanic acid oral	1026	67.2
Cephalosporin (1st generation) parenteral (IV)^a^	180	11.8
Cephalosporin (1st generation) oral^b^	130	8.5
Cephalosporin (3rd generation; SC)^c^	13	0.8
Fluoroquinolone parenteral (SC, IV)^d^	65	4.2
Fluoroquinolone oral^d^	52	3.4
Metronidazole parenteral (IV)	75	4.9
Metronidazole oral	88	5.7
Penicillin narrow spectrum oral	2	0.1
Penicillin extended spectrum parenteral (IV)^e^	46	3
Penicillin extended spectrum oral^e^	18	1.2
Ticarcillin/clavulanic acid parenteral (IV)	1	0.06
Trimethoprim—sulfonamide parenteral (SC)	2	0.1
Trimethoprim—sulfonamide oral	15	0.9
Tetracycline oral^f^	4	0.2
Lincosamide oral^g^	16	1
Topical antimicrobial	44	2.9

^a^ Cefazolin.

^b^ Cephalexin.

^c^ Cefovecin.

^d^ Enrofloxacin.

^e^ Amoxicillin, ampicillin.

^f^ Doxycycline.

^g^ Clindamycin.

Prescribed dosages for AMC, cephalosporins and enrofloxacin were compared to recommended dosages.^25^, ^26^ Oral and parenteral AMC were prescribed at dosages less than 12.5 mg/kg in 13.4% (220/1647) of prescriptions. Cefazolin and cephalexin were both prescribed at dosages less than 22 mg/kg in 26.1% (81/310) of prescriptions and cefovecin were prescribed lower than 8 mg/kg in 7.7% (1/13) of dogs. Enrofloxacin was prescribed at lower than the recommended 5 mg/kg dose in 11.1% (13/117) of dogs.

The median frequency and duration of oral dosing AMC and cefazolin was twice a day for 7 days (Table 4). Cefovecin was usually given as a 1‐off dose which has a therapeutic effect for 14 days. The median frequency of oral and parenteral dosing for enrofloxacin was 1 dose every 24 hours and the median duration was 3 days for the parenteral and 7 days for the oral formulation (Table 4).

**TABLE 4:** Antimicrobial frequency and duration of administration prescribed to treat 1526 dog bite events which presented for treatment between 1999 and 2019

Antimicrobial	Frequency (dose per day)			Duration (d)		
	Median	Minimum	Maximum	Median	Minimum	Maximum
Amoxicillin‐clavulanic acid parenteral (SC)	1	1	5	1	1	14
Amoxicillin‐clavulanic acid oral tablets	2	1	3	7	1	56
Amoxicillin‐clavulanic acid oral liquid	2	1	3	8.5	3	30
Cefazolin (IV)	3	1	8	2	1	12
Cephalexin oral tablets	2	1	4	7	1	22
Convenia (SC)	1	1	2^a^	1	1	21
Fluoroquinolone parenteral (SC, IV)	1	1	2	3	1	15
Fluoroquinolone oral	1	1	2	7	3	20

^a^ Two doses 14 days apart.

### 3.4 Antimicrobial susceptibility results

Culture and susceptibility testing were performed on 1.8% (27/1526) DBW and results were available for 16 dog bite events. Of these 6.3% (1/16) had grade 3 wounds, 75% (12/16) had grade 4 wounds and 18.7% (3/16) had grade 5 wounds. Complications were recorded in 12.5% (2/16) events (1 had purulent discharge and the other an abscess). No complications were recorded in 87.5% (14/16) dog bite events, however in 42.8% (6/14) of the events from all 3 hospitals, no recheck was performed. No association between complications and wound severity could be identified due to the small sample size. Twenty bacterial isolates were cultured with 14 different organisms identified (Table 5). Antimicrobial susceptibilities of all isolates cultured were separated into gram‐negative (n = 12) and gram‐positive (n = 8). Of the gram‐negative isolates, 83.3% (10/12) were susceptible to at least 1 antimicrobial of low‐importance. Of the isolates resistant to low‐importance antimicrobials, 1 (8.3%) was susceptible to all medium‐importance antimicrobials and the other was only susceptible to 1 antimicrobial of high‐importance. Of the gram‐positive isolates, 87.5% (7/8) were susceptible to at least 1 antimicrobial of low‐importance. The isolate which was resistant to the low‐importance antimicrobials, was susceptible to all medium‐importance antimicrobials. Six of the identified isolates were multidrug resistant (defined as resistant to at least 1 agent in 3 or more antimicrobial categories, excluding intrinsic resistance): 3 *Pseudomonas* spp., 2 *Enterococcus* spp. and 1 *Escherichia hermannii*.^27^ All of the MDR isolates were cultured between the years 2004 and 2016, with 50% cultured between 2004 and 2009.

**TABLE 5:** Bacteria cultured from 16 dogs which presented with dog‐to‐dog bite wounds for treatment between 1999 and 2019

Pathogen cultured	Number of dogs	
	n	%
No growth	2	12.5
Very light, mixed growth with no predominant organism	1	6.2
Gram negative bacillus	2	14.2
*Enterococcus* spp.	2	14.2
*Enterococcus faecium*	1	6.2
*Enterobacter cloacae*	1	6.2
*Staphylococcus pseudintermedius*	2	14.2
*Staphylococcus aureus*	1	6.2
*Streptococcus canis* (Group G)	2	14.2
*Pseudomonas* sp.	1	6.2
*Pseudomonas aeruginosa*	3	18.7
*Escherichia coli*	1	6.2
*Escherichia hermannii*	1	6.2
*Proteus mirabilis*	1	6.2
*Pasteurella* sp.	1	6.2
*Pasteurella multocida*	1	6.2

### 3.5 Clinic and year of presentation

**FIGURE 4:**
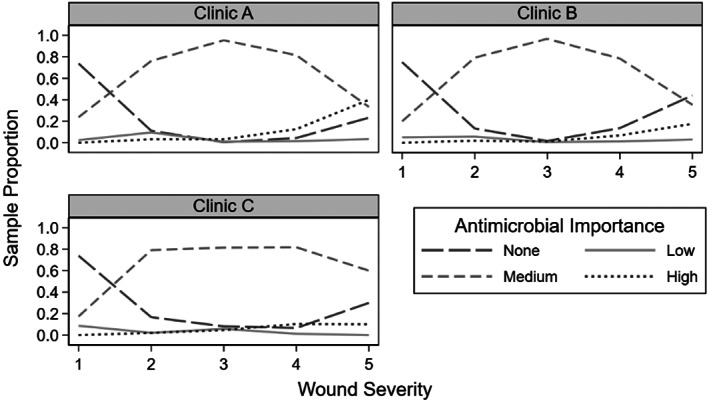
Antimicrobial importance class vs wound severity for the 3 different clinics for 1436 dogs presenting for treatment of dog‐to‐dog bite wounds from 1999 to 2019. Clinic A, urban, specialist referral/general practice hospital; Clinic B, semirural, specialist referral/general practice hospital; Clinic C, semirural, mixed and general practice clinic

**FIGURE 5:**
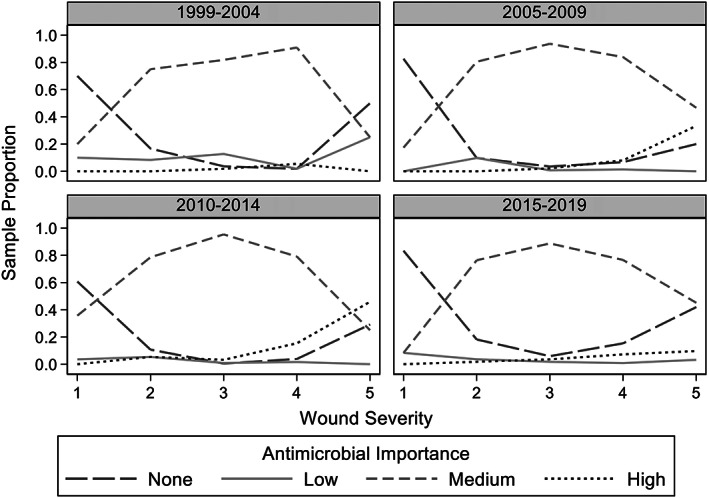
Antimicrobial importance class by year vs wound severity for 1436 dogs presenting for treatment of dog‐to‐dog bite wounds from 1999 to 2019

**FIGURE 6:**
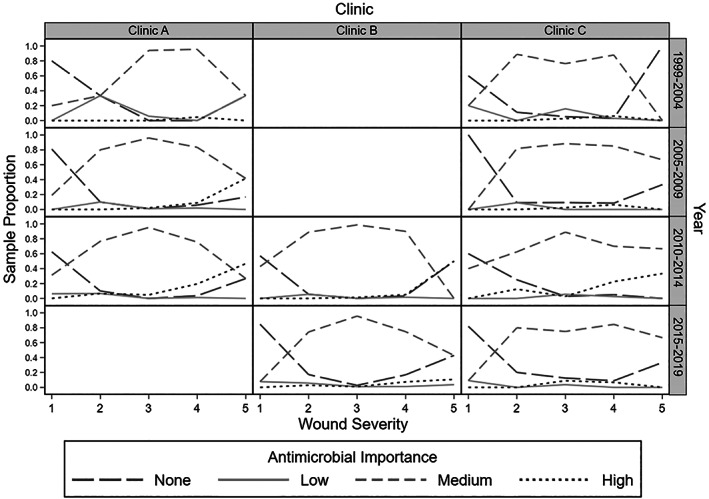
Antimicrobial importance class by year and clinic vs wound severity for 1436 dogs presenting for treatment for dog‐to‐dog bite wounds from 1999 to 2019. Clinic A, urban specialist primary accession practice; Clinic B, semirural, specialist referral/general practice; Clinic C, semirural mixed practice

Clinic A saw 37.6% (540/1436) of the dogs, Clinic B saw 36.3% (521/1436) and Clinic C saw 26.1% (375/1436; Table 2 and Figure 4). Clinic A and B were not operational between 2015‐2019 and 1999‐2004, and 2005‐2009 respectively. The years 2010‐2014 and 2015‐2019 had the most dogs presenting for DBW with 481 and 484 dogs, respectively (Figures 5 and 6). Only 127 dogs presented at the earliest time‐period of 1999‐2004.

### 3.6 Reexamination, complications, and death

A repeat examination was performed on 21.2% (323/1526) of DBW events, 59.9% (914/1526) were lost to follow‐up and 18.9% (289/1526) were referred back to their primary care veterinarian. Of the dogs in which reexamination was performed, no complications associated with infection occurred in 86.9% (281/323). Forty‐two dogs had recorded complications consistent with possible infection including: purulent discharge in 33.3% (14/42), wound dehiscence in 40.0% (13/42), abscessation in 23.8% (10/42), inflammation of the site in 9.5% (4/42) and septic peritonitis in 2.4% (1/42). Of the dogs with complications, 16.6% (7/42) were grade 3 wounds and 66.6% (28/42) were grade 4. Of these 42 dogs, inappropriately low doses of antimicrobials were prescribed in 26.2% (11/42) of dogs. Complications included purulent discharge (3/11), abscessation (3/11), wound dehiscence (2/11), inflammation (2/11) and septic peritonitis (1/11). The most common inappropriately low dosed antimicrobials in these dogs were AMC (<12.5 mg/kg PO, n = 6) and cephalexin (<22 mg/kg PO, n = 4), followed by AMC (parenteral, n = 2), metronidazole (<10 mg/kg PO, n = 2) and enrofloxacin (<5 mg/kg parenteral, n = 1). Of the 275 dogs which had no recorded complication, 248 ERs had sufficient information to determine dosage, of which 25.4% (63/248) were prescribed an inappropriately low dose. There was statistically significant association between appropriateness of antimicrobial dose and complication rate $\left(\chi^{2}\left(1\right)=0.01,P=.09\right)$.

The overall case fatality rate directly related to the DBW was 7.2% (110/1526). One hundred one dogs were euthanized and 9 died of cardiopulmonary arrest. Records did not elucidate if euthanasia was performed due to financial reasons or extent of injuries and perceived poor prognosis, however 13 dogs were euthanized for behavioral reasons.

### 3.7 Model for association between higher importance antimicrobials and wound severity, clinic, and year

When comparing the initial dataset of all dogs compared to the analytic dataset used for inferential modeling, differences in antimicrobial importance class, number of antimicrobials prescribed and wound severity grades was found (*P* < .001; Table 2). Neuter status was also significantly different (*P* < .001); this could be due to an association between neuter status and death in this sample $\left(\chi^{2}\left(1\right)=20.0,P<.001\right)$ where 11.4% of entire animals died or were euthanized, compared to 5.0% of neutered animals. For year, the greatest contribution to difference was between 2015‐2019, which is likely due to exclusion of dogs enrolled in a concurrent prospective study.^14^ Season was associated with death with 10.1% (37/368) of DBWs in summer resulting in euthanasia or arrest, compared to 4.5% (19/420) in winter $\left(\chi^{2}\left(3\right)=9.2,P=.03\right)$. There was no difference between the datasets for age, sex, weight, time of attack and duration of hospitalization.

A partial proportional odds model of the analytic dataset was used to estimate the multivariate adjusted OR of prescribing an antimicrobial of higher importance (Table 6). There was an association between wound severity of the DBW and importance of antimicrobial prescribed (*P* < .001). As the wound severity increased by 1 grade, there was an estimated 97% (95% CI: 42%‐173%) increase in the odds of a higher antimicrobial class being prescribed (Table 6 and Figure 2).

**TABLE 6:** Odds ratios with 95% confidence interval (CI) and *P* values for the partial proportional odds model for prescribing an antimicrobial of higher importance

	Odds ratio	95% CI	*P* value
Wound severity grade	1.97	1.42‐2.73	**<.001** *
Age (y)	0.96	0.90‐1.02	**.19**
Sex			
Male	Baseline		
Female	0.95	0.62‐1.44	**.81**
Neuter status			
Entire	Baseline		
Neutered	0.89	0.55‐1.44	**.63**
Weight			**.87**
<10 kg	Baseline		
10‐25 kg	0.99	0.57‐1.77	.99
25+ kg	0.81	0.44‐1.48	.49
Unknown	0.96	0.51‐1.82	.91
Clinic			**<.001** *
Clinic C	Baseline		
Clinic B (low‐importance vs high‐importance antimicrobials)	1.21	0.44‐3.32	.72
Clinic B (medium‐importance vs high‐importance antimicrobials)	0.18	0.08‐0.41	<.001*
Clinic A	1.10	0.61‐1.98	.75
Year of consultation			**<.001** *
1999‐2004	Baseline		
2005‐2009 (low‐importance vs high‐importance antimicrobials)	5.73	2.00‐16.40	.001*
2005‐2009 (medium‐importance vs high‐importance antimicrobials)	2.11	0.86‐5.18	.10
2010‐2014	5.23	2.41‐11.35	<.001*
2015‐2019	4.23	1.81‐9.86	.001*
Type of therapy			
Monotherapy	Baseline		
Polytherapy	18.93	9.61‐37.33	**<.001** *
C&S performed			
Not performed	Baseline		
Performed	1.93	0.57‐6.53	**.29**
Time of attack			**.71**
<8 h	Baseline		
≥8 h	0.76	0.38‐1.49	.42
Unknown	0.86	0.49‐1.50	.59
Season of attack			**.44**
Spring	Baseline		
Summer	1.25	0.69‐2.27	.46
Autumn	0.85	0.44‐1.48	.62
Winter	1.34	0.51‐1.82	.34
Duration of hospitalization (days)	1.15	1.05‐1.27	**.004** *

* Significant at the .05 level. The *P* values for the individual levels of the categorical variables compared to the baseline are given, in addition to a bolded *P* value for all levels of that categorical variable combined.

The number of antimicrobials (monotherapy vs polytherapy) was associated with the antimicrobial importance class prescribed (*P* < .001). There was an estimated 18.9 times increase (95% CI: 9.6‐37.3) in the odds of an antimicrobial of higher importance being prescribed when polytherapy was used rather than monotherapy (Table 6). There was an association between duration of hospitalization and antimicrobial importance class prescribed (*P* = .004). For each additional day of hospitalization, there was an estimated 15% (95% CI: 5%‐27%) increase in the odds of an antimicrobial of higher importance being prescribed (Table 6).

An association between clinic and level of antimicrobial importance prescribed was detected (*P* < .001). Clinic C dispensed more high‐importance rather than medium‐importance antimicrobials than Clinic B (*P* < .001). The estimated odds for Clinic B prescribing high‐importance vs medium‐importance antimicrobials was 82% lower than Clinic C (95% CI: 59%‐92%), after adjusting for other variables, including year category (Table 6).

Differences between year category and the level of antimicrobial importance class prescribed were detected (*P* < .001). Compared to 1999‐2004, the estimated odds of a higher importance antimicrobial being prescribed was 5.2 times (OR 5.2, 95% CI: 2.4‐11.4) higher for the period 2010‐2014 (*P* < .001) and 4.2 times (OR 4.2, 95% CI: 1.8‐9.9) higher for the time period 2015‐2019 (*P* = .001; Table 5). There was an estimated 5.7 times (OR = 5.7, 95% CI: 2.0‐16.4) increase in the OR of prescribing an antimicrobial of high‐importance than low (*P* = .001) during period 2005‐2009 than 1999‐2004, and 2.1 times (OR = 2.1, 95% CI: 0.9‐5.2) increase in the odds of prescribing medium‐importance compared to high‐importance antimicrobial, although this difference was not significant (*P* = .10). Age, sex, neuter status, weight category, C&S testing, time of attack to presentation and season of attack, were not associated with the level of antimicrobial importance prescribed.

## 4 DISCUSSION

Culture and susceptibility testing were rarely performed on cases of DBW. Antimicrobials were considered unnecessary in 7.3% of dogs mostly with low severity (grades 1 and 2) wounds. Empirical antimicrobial treatment was administered to 88% of dogs, with medium‐importance antimicrobials AMC and first‐generation cephalosporins commonly prescribed. However, 13% of dogs prescribed AMC and 26% of dogs prescribed first‐generation cephalosporins were underdosed. A 97% increase in the odds of a prescription of a higher antimicrobial importance class occurred with each wound severity grade. Antimicrobials of higher importance were more likely to be dispensed in conjunction with antimicrobial polytherapy and increased duration of hospitalization. Semirural mixed/general practice (Clinic C) prescribed antimicrobials of a higher importance more frequently than semirural, specialist referral/general practice hospital (Clinic B). Antimicrobials of high‐importance were more commonly dispensed between 2005‐2009, 2010‐2014 and 2015‐2019 than between 1999‐2004.

The most common signalment of DBW cases was middle‐aged, medium sized, pure breed desexed female dogs. Previous studies found on average, small breed (<10 kg), entire male dogs presented more commonly for DBW.^1^, ^4^, ^9^, ^12^, ^28^, ^29^ Authors suggested hormonal and territorial triggers in entire male dogs coupled with the vulnerability of smaller dogs predisposed to more severe injury necessitating medical attention.^1^, ^4^, ^28^, ^29^ In our study, the antimicrobial use analytic data set had more desexed dogs than the initial dataset (*P* < .001). Neuter status and deaths were associated, with a greater proportion of entire dogs euthanized or died. Although not previously reported, it might be due to wound severity or entire dogs being deemed more aggressive by owners prompting euthanasia on behavioral grounds. However, a study investigating dog bites in humans found no associated between neuter status and the likelihood of inflicting a DBW.^30^ Similar to other studies, most dogs presented for veterinary attention less than 8 hours post injury.^1^, ^4^, ^5^, ^11^, ^29^

Eighty‐eight percent of DBW were prescribed antimicrobials. AMC was the most prescribed antimicrobial at 73%, followed by first‐generation cephalosporins with 8% of dogs prescribed oral cephalexin at discharge. This was similar to a recent study of thoracic DBW in which AMC and first‐generation cephalosporins were prescribed at 71% and 14%, respectively.^9^ The BSAVA and AIDAP guidelines recommend AMC as first‐line treatment for DBW, however APCAH guidelines recommend using amoxicillin.^17^, ^18^, ^19^ The Swedish Veterinary Association (SVA) recommends ampicillin or amoxicillin as a first‐line of treatment excluding cases of known or suspected staphylococcal infection, in which cephalosporins or clindamycin are recommended.^31^ Irrespective of conflicts in first‐line antimicrobial recommendations, the use of a lower importance antimicrobials such as amoxicillin as a first‐line over a medium‐importance antimicrobial, adheres to the principles of antimicrobial stewardship. Most veterinarians in this study are following the guidelines, however continuing education is recommended.

Our study found an association between the use of high‐importance antimicrobials and increasing wound severity, antimicrobial polytherapy and hospital duration. This was anticipated as more severe wounds, such as grade 5 wounds, would require intensive treatment and longer hospitalization. In these cases, especially with risk or clinical indication of sepsis, broad‐spectrum polytherapy (AMC and enrofloxacin) is often used while awaiting results of C&S testing. Often antimicrobials of high‐importance are prescribed, with the intention to de‐escalate upon either results of C&S testing and clinical improvement. However, of the dogs in our study which received a third‐generation cephalosporin (13) or a fluoroquinolone (117), only 7.6% (n = 10) had C&S testing performed. Therefore, 92% of high‐importance antimicrobials were used empirically. Antimicrobials of high‐importance are considered essential for the treatment of infections in people, as critical or last‐line antimicrobials, and are not recommended for prophylactic use in human medicine.^20^, ^23^ Use of fluoroquinolones without susceptibility testing is contrary to the World Health Organizations international guidelines for the prudent use of antimicrobials in animals.^23^ Label statements for both enrofloxacin and cefovecin stipulate use only after C&S testing indicates no suitable alternatives (Enrofloxacin, Dechra Veterinary Products Pty Ltd., Somersby, NSW, Australia; Convenia, Zoetis Inc, Kalamazoo, MI, USA). In our study, 7.6% of DBW cases received a fluoroquinolone. Studies assessing prescribing of antimicrobial by Australian veterinarians found enrofloxacin was dispensed in 3.2% to 18% of cases.^32^, ^33^ In New Zealand, fluoroquinolones were prescribed in 12% of cases and of these, 53% had no C&S testing performed.^34^ Similar to our study, an association between the use of fluoroquinolones and antimicrobial polytherapy was found.^34^

Clinic C (semirural/mixed practice) had a higher rate of prescribing antimicrobials of higher importance compared to Clinic B (specialist referral/general practice hospital). Although the referral hospital was likely to be treating more severe cases of DBW, specialist veterinarians could adhere to the principles of antimicrobial stewardship more closely because of more extensive training. Alternatively, nonspecialist veterinarians could be more concerned about ensuring a successful outcome after a single consultation due to owner financial limitations. Australian companion animal veterinarians from regional areas used less antimicrobials compared to major cities, but the use of high‐importance antimicrobials was higher in outer regional areas and major cities compared to inner‐regional areas.^33^ Although there was no difference in the proportion of high‐importance antimicrobials prescribed, emergency and referral centers prescribed more antimicrobials than general practice as routine examinations and vaccinations rarely require antimicrobials.^33^ Another study of Australian companion animal veterinarians found no differences in the prescription of antimicrobials of high‐importance, socioeconomic variables, postcode or graduation year of the prescribers.^32^

This study found higher importance antimicrobials were used more commonly between 2005‐2009 and 2010‐2014 compared to 1999‐2004, especially for grade 5 wounds. Increasing use of high‐importance antimicrobials over the study likely coincided with their availability. In Australia, enrofloxacin was registered for veterinary use in 1995 and cefovecin in 2008.^35^ Records for the 3 clinics showed enrofloxacin was first ordered in 2002 and cefovecin in 2008.

Only 1.8% of wounds had C&S testing performed, likely due to perceived increase in costs. However routine use of C&S testing and subsequent de‐escalation of empirical enrofloxacin therapy in higher severity wounds, is likely financially advantageous with no reduction in treatment success. Determining the organisms present in DBW was an initial aim of this study. However, with only 20 bacterial isolates cultured, it is difficult to draw any conclusions. The most isolated bacteria were *Pseudomonas aeruginosa* (21.4%), *Staphylococcus pseudintermedius* (14.2%), *Enterococcus* spp. (14.2%) and *Streptococcus canis* (Group G; 14.2%). This is similar to previous DBW studies which commonly cultured *S. pseudintermedius*, *Enterococcus* spp., *Escherichia coli*, and *Pasteurella multocida*.^4^, ^5^, ^7^, ^9^, ^10^, ^11^, ^12^, ^13^

It is unknown if C&S testing was performed at initial presentation or after clinical deterioration or treatment failure. Complications were only recorded in 2/16 cases which had C&S performed. This suggests samples were taken at initial presentation but the high rate of MDR bacteria isolated (35%, 7/20) suggests that C&S testing could have been performed due to poor response to initial antimicrobial therapy. While the rate of MDR is higher than previously reported (6%‐19%^10^, ^11^), low numbers and retrospective nature of this study make conclusions difficult. As 2 of the clinics were specialist/referral clinics, there could have been an overrepresentation of dogs with more severe injuries, signs of existing infection and previous antimicrobial use. Performing C&S testing is recommended in the prescribing guidelines for treatment of penetrating wounds.^17^, ^18^, ^19^, ^36^ Evidence‐based approach to drug selection likely reduces treatment costs by reducing improper drug selection and unnecessary use of expensive antimicrobial drugs, leading to prolonged treatment and increased morbidity and death.^37^ It is important to note that in vitro susceptibility results might not represent in vivo effects and antimicrobial treatment should be tailored to individual dog requirements.

All DBW are considered contaminated with more than 1 microbe.^20^ One study found 84% of DBW cultured positive, however, only 17% to 20% were clinically infected.^36^ Cultured samples taken at presentation of clinically noninfected wounds do not predict whether a wound will become infected, the potential infectious organism or the correct antimicrobial therapy.^2^, ^7^, ^12^ Infected wounds are more likely to have positive cultures and antimicrobial therapy is warranted.^4^, ^12^ Antimicrobial therapy aims to control bacterial growth and enable host responses to contain or eliminate the bacteria responsible for disease.^36^ Antimicrobials are not a substitute for appropriate cleaning or surgical management of DBW.^2^, ^7^, ^36^

Complications consistent with possible infection occurred in 15% of dogs which presented for reexamination, which is slightly higher than 7% to 12% previously.^7^, ^28^, ^38^ Of dogs with complications, 29% (n = 12) were prescribed inappropriately low doses of antimicrobials and only 4 of these dogs had C&S testing performed. This suggests that inappropriately low doses of antimicrobials prescribed could be a contributing factor to the development of infection. Frequency of antimicrobial underdosing (AMC: 13%, first‐generation cephalosporins: 26%, enrofloxacin 11%) in our university teaching hospitals is concerning. Guidelines (BSAVA, APCAH, SVA) do not provide dose rates for recommended first‐line antimicrobials, however AIDAP guidelines recommend AMC at 12.5 mg/kg.^17^, ^18^, ^19^, ^25^, ^26^, ^31^ Of the dogs prescribed AMC and first‐generation cephalosporins, 7% and 9.6% respectively received a single parenteral injection with no ongoing course of oral administration. The reasons for not receiving an ongoing course of antimicrobials are unknown, however the use of a single, 1‐off short acting antimicrobial could have been associated with surgical prophylaxis in contaminated wounds. Improved education of students and veterinary practitioners is needed.

The main limitations of this study are due to its retrospective nature. Therefore, misclassification bias might exist, such as wound severity grades, as these were determined based on the examination of text data. Missing data from the medical records could have resulted in selection bias, especially in cases where reexamination and follow up records were inaccessible, such as those discharged to a referring veterinarian. Furthermore, the data from the 3 hospitals were not over the same time period, although the time periods overlapped and year category was accounted for in the statistical model. There is a potential lack of generalizability of the results; although data was obtained from 3 hospitals, 2 semirural and 1 urban, the results might not reflect cohorts in different states or countries. Due to the small number of C&S tests performed, the small number of isolates and changing antimicrobial breakpoints over time, no conclusions could be made on the appropriateness of the guidelines in this study sample. The ASTAG antimicrobial importance ratings were also used over the WHO guidelines. This has a potential to limit generalizability however, the ASTAG ratings were created and revised with an awareness of WHO guidelines. The WHO guidelines also recognize that implementation at the national level requires national considerations.

## 5 CONCLUSIONS

This study found that AMC is the most commonly prescribed antimicrobial for DBW in SEQ. C&S testing is rarely performed, despite this, the increasing use of antimicrobials of high‐importance over the years is concerning, especially as published human and veterinary guidelines recommend they be reserved as last‐line therapy. More education is needed to ensure great compliance with prescribing guidelines for DBW; ensure appropriate dosing of antimicrobials; minimize use of high‐importance antimicrobials and increasing the frequency of C&S testing. No antimicrobials were used in 74% of grade 1 wounds and 32% of grade 2 wounds. Therefore, it is the authors' recommendation that no antimicrobials be used in low grade DBW, however a prospective placebo controlled blinded study would be ideal to confirm this. Further prospective research is required to determine appropriate empirical antimicrobial therapy for DBW, and to understand further Australia‐wide trends for treatment of DBW, and frequency and effects of underdosing of antimicrobials.

## CONFLICT OF INTEREST DECLARATION

Authors declare no conflict of interest.

## OFF‐LABEL ANTIMICROBIAL DECLARATION

Authors declare no off‐label use of antimicrobials.

## INSTITUTIONAL ANIMAL CARE AND USE COMMITTEE (IACUC) OR OTHER APPROVAL DECLARATION

Approval for the use of medical records was granted by the University of Queensland Animal Ethics Committee (Approval number: ANRFA/SVS/298/19).

## HUMAN ETHICS APPROVAL DECLARATION

Authors declare human ethics approval was not needed for this study.
